# Seroepidemiology of dengue and chikungunya fever in patients with rash and fever in Iran, 2017

**DOI:** 10.1017/S0950268820000114

**Published:** 2020-02-26

**Authors:** Forough Tavakoli, Farhad Rezaei, Nazanin Zahra Shafiei-Jandaghi, Azadeh Shadab, Talat Mokhtari-Azad

**Affiliations:** 1 Virology Department, School of Public Health, Tehran University of Medical Sciences, Tehran, Iran; 2 National Reference Laboratory for Measles and Rubella, School of Public Health, Tehran University of Medical Sciences, Tehran, Iran

**Keywords:** Arboviruses, chikungunya virus, dengue fever, virology (human) and epidemiology

## Abstract

After the mass campaign of Measles and Rubella vaccination in 2003 in Iran, the cases of measles and rubella infection decreased but still, the cases of rash and fever were reported. It is worth noting that some other viral infections show signs similar to measles and rubella such as some arboviruses. Considering the epidemic outbreak of arbovirus infections in countries neighbouring Iran, we performed this study to estimate the possibility of chikungunya and dengue fever among measles and rubella IgM negative patients presenting with rash and fever from December 2016 to November 2017 in the National Measles Laboratory at Tehran University of Medical Sciences. Serum samples were selected at random from patients from eight provinces. The presence of DENV IgM and CHIKV IgM was examined by enzyme-linked immunosorbent assay. Of the 1306 sera tested, 210 were CHIKV seropositive and 82 were dengue seropositive. Statistical analysis demonstrated a significant increase in the CHIKV IgM antibody seropositivity rate in Kerman (OR = 2.07, 95% CI: 1.10–3.92; *P* = 0.024) and Fars (OR = 1.77, 95% CI: 1.06–2.93; *P* = 0.027). The DENV and CHIKV seropositivity rate in summer is higher than in other seasons (*P* < 0.01). Our seropositive samples suggest possible CHIKV and DENV infection in Iran. It is likely that these viruses are circulating in Iran and there is a need to study vector carriage of these two viruses.

## Introduction

Measles and rubella infections in Iran decreased due to the mass campaign of measles and rubella vaccination in 2003 [1] but still, there are suspected cases. National Measles Laboratory (NML) in Tehran University of Medical Sciences receives samples for about 6000 suspected cases of measles and rubella in 1 year. Fortunately, the Measles/Rubella incidence rate is less than expected. It is worth noting that some other viral infections show similar signs with measles and rubella such as some arboviruses [2]. Over the past decade, a considerable global emergence/re-emergence of arboviral diseases occurred [3]. Arboviruses cause some of the most important emerging infectious diseases which are associated with fever and rash in tropical and subtropical regions [4, 5]. Of these viruses, chikungunya virus (CHIKV) belonging to the alphavirus genus in the Togaviridae family[6] has re-emerged in Africa, the Indian Ocean islands, South and Southeast Asia, with an emergence seen in the Americas, the Pacific and Europe due to the transatlantic and transpacific migration of patients [7]. Another arbovirus is dengue virus (DENV), an RNA virus that belongs to the Flaviviridae family with four closely related but antigenically different DENV serotypes 1–4 [8]. It is responsible for about 400 million estimated infections annually resulting in asymptomatic or mild dengue fever to dengue haemorrhagic fever [9]. Currently, DENV infection is a major health threat and the most important human arbovirus disease in both the tropics and subtropical regions [10, 11]. The CHIKV and DENV transmission between humans occurs through different *Aedes* species including *Aedes aegypti* and *Aedes albopictus* mosquitoes which are distributed throughout the world [4, 12]. Signs of disease in CHIKV and DENV infections are associated with arthralgia, fever, rash and also other manifestations such as muscle pain, headache, nausea and fatigue [4]. In Iran, the epidemiology of DENV and CHIKV remains poorly understood. Iran is a country located in West Asia. Iran is bordered to the east by Afghanistan and Pakistan and to the west by Turkey and Iraq, countries where epidemics and endemics of arboviruses including CHIKV and DENV have occurred [13, 14].

Therefore, Iran is strategically placed at a remarkable risk zone for these arboviral diseases. In addition, the introduction of exotic vectors into Iran is possible [15]. Active entomological surveillance of *Ae. albopictus* and *Ae. aegypti* was carried out in spring, summer and winter, 2008–2014 in eight provinces [15] following the first dengue case report in Iran in 2008 [16]. *Ae. albopictus* larvae were collected in the Sistan & Baluchestan province bordering Pakistan in 2009 [15]. In 2013, *Ae. albopictus* adult mosquitoes were also detected in a coastal location neighbouring Chabahar in the same province [15]. *Ae. albopictus* is regarded as a conspicuously menacing and versatile species that inhabits both temperate and tropical climate regions [17, 18]. Since this vector has been detected in different parts of Sistan & Baluchestan, there is a possibility of dengue fever and chikungunya fever in southeast Iran. At present, there are just a few studies from Iran on dengue fever in blood donors [19] and on patients who tested negative for Crimean-Congo Haemorrhagic Fever in Sistan & Baluchestan province [20] and there is no report from Iran on dengue fever in patients with rash and fever showing non-specific febrile illness. Between 2017 and 2018, an investigation was cariied out in the Sistan and Baluchistan Province on alleged CHIK patients. Laboratory results from qRT-PCR or ELISA tests performed revealed a quarter (25.1%) of the samples tested positive [21]. In addition, another study in 2017 depicted the southernmost regions as areas at a high risk of *Ae. albopictus* establishment [17]. Hence, this study was implemented with the aim of assessing the serology of chikungunya and dengue fever in suspected patients.

## Methods

### Serum specimens

NML in the School of Public Health, Tehran University of Medical Sciences, every year, receives about 6000 samples from suspected cases of measles and rubella, and laboratory diagnosis was made using IgM capture enzyme-linked immunosorbent assay (ELISA) for measuring IgM anti-measles and IgM anti-rubella antibody.

Serum samples were collected randomly from eight provinces (Table 1) from December 2016 to November 2017. The patients had fever and rash, but the sera were negative for measles and rubella IgM. We have studied the areas where active entomological surveillance of *Ae. albopictus* and *Ae. aegypti* was carried out in spring, summer and winter, 2008–2014 in those regions.

**Table 1.** Anti-CHIKV and anti-dengue seropositivity rates in studied samples

	Participants (*n* = 1306)	Anti-CHIK V positive (*n* = 210)	Anti-CHIK V negative (*n* = 1096)	*P*-Value	Odds ratio	0.95 CI	Anti-DENV positive (*n* = 82)	Anti-DENV negative (*n* = 1224)	*P*-Value	Odds ratio	0.95 CI
Gender											
Male	701	109	592				33	668			
Female	605	101	504	0.572	1.093	0.804–1.485	49	556	0.015	1.77	1.11–2.83
Season											
Summer	255	53	202	0.000			28	227	0.000		
Fall	411	71	340	0.178	0.75	0.50–1.13	35	376	0.263	0.734	0.42–1.26
Winter	243	65	178	0.121	1.39	0.91–2.13	11	232	0.008	0.374	0.18–0.77
Spring	397	21	376	0.000	0.229	0.13–0.39	8	389	0.000	0.15	0.06–0.35
Age groups(year)											
Infant	629	111	518	0.554			50	579	0.14		
Youth	566	87	479	0.424	0.87	0.63–1.20	29	537	0.063	0.62	0.38–1.02
Adult	107	12	95	0.177	0.63	0.33–1.22	3	104	0.092	0.35	0.10–1.18
Elderly	4	0	4	0.999	0.00	0.00	0	4	0.999	0.00	0.00
Province											
Khuzestan	305	28	277	0.066			16	289	0.803		
Fars	294	64	230	0.027	1.77	1.06–2.93	21	273	0.611	0.831	0.40–1.69
Kerman	100	23	77	0.024	2.07	1.10–3.92	6	94	0.556	0.74	0.27–2.01
Ilam	23	5	18	0.122	2.40	0.791–7.28	3	20	0.233	2.31	0.583–9.16
Hormozgan	76	14	62	0.549	1.25	0.60–2.59	5	71	0.862	0.908	0.30–2.67
Bushehr	56	7	49	0.895	0.94	0.37–2.33	3	53	0.688	0.76	0.20–2.81
Sistan & Baluchestan	142	24	118	0.492	1.24	0.67–2.30	10	132	0.519	0.753	0.31–1.78
Khorasan-e-Jonubi	310	45	265	0.869	1.04	0.61–1.77	18	292	0.285	0.67	0.32–1.39

Iran is a country with an area of 1,648,000 km^2^ located in the Middle East with a diverse climate [22]. Among the studied provinces during 2017, Ilam and Sistan & Baluchestan had the highest (398.8 mmHg) and the lowest (28.7 mmHg) average annual rainfall respectively [23]. Khuzestan had the highest (50.4 °C) maximum temperature and Ilam had the lowest (−8.9 °C) minimum temperature [24].

### Enzyme-linked immunosorbent assay

The presence of DENV IgM and CHIKV IgM were examined by a commercial Semiquantitative IgM ELISA kit according to the manufacturer's instructions (EUROIMMUN Anti-CHIKV/IgM) and (EUROIMMUN Anti-DENV/IgM; PerkinElmer, Lübeck, Germany).

### Statistical analyses

Statistical analyses were performed using the SPSS software, version 16. Fisher's exact test was performed for the evaluation of qualitative variables including sex, province and season. In order to explain the relationship between dengue and chikungunya infection and independent variables, logistic regression was performed. *P*-Value <0.05 was considered significant.

## Results

In this study, serum samples from 1306 patients from eight provinces were tested.

The overall CHIKV and DENV IgM seropositivity was 16.07% (210/1306) and 6.27% (82/1306) respectively. A summary of the characteristics of the study of all the patients was presented (Table 1).

**Fig. 1.**
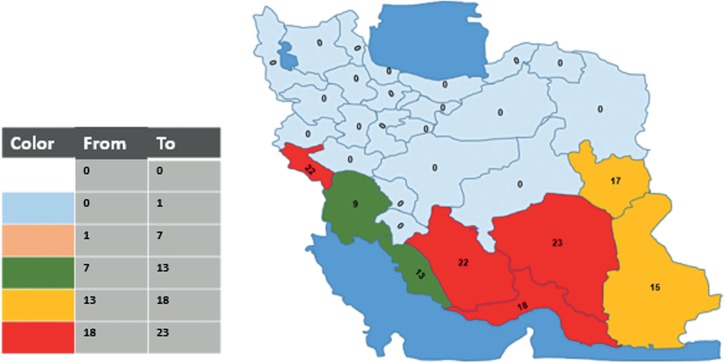
Map showing the CHIKV Seropositivity rate in study areas in Iran, 2017.

**Fig. 2.**
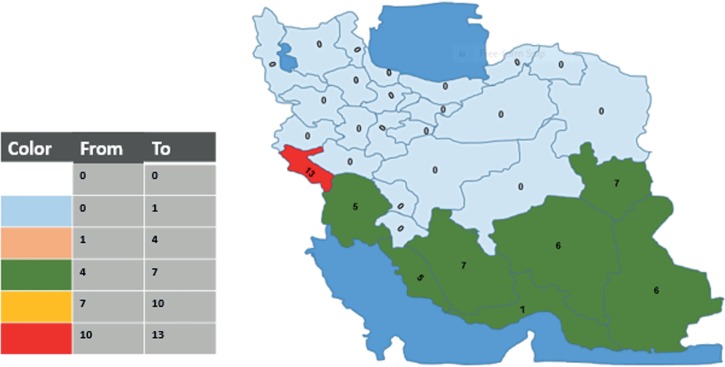
Map showing the DENV Seropositivity rate in study areas in Iran, 2017.

The rate of chikungunya seropositivity among patients from the eight provinces ranged from 9.18% in Khuzestan (28/305) to 23% in Ilam (23/100) (Fig. 1). The rate of dengue seropositivity among patients from the eight provinces ranged from 5.24% in Khuzestan (16/305) to 13.04% in Ilam (3/23) (Fig. 2). The highest rate of chikungunya seropositivity was found in Kerman, Fars and Ilam, and the highest rate of dengue seropositivity was found in Ilam, Fars and Khorasan-e-Jonubi. Logistic regression analysis showed a significant increase in the CHIKV IgM antibody seropositivity rate in Kerman (OR = 2.07, 95% CI: 1.10–3.92; *P* = 0.024) and Fars (OR = 1.77, 95% CI: 1.06–2.93; *P* = 0.027).

The patients were divided into four age groups comprising less than or equal to 1 year (infants), 2–18 years (youths), 19–64 years (adults) and more than 64 years (elderly). The frequency distribution of the various age groups encompassing groups 1 to 4 stated above was 48.2% (629/1306) for infants, 43.3% (566/1306) for youths, 8.2% (107/1306) for adults and 0.3% (4/1306) for elderly respectively. The mean (±s.d.) age of the studied cases was 6.05 (±9.536). The highest anti-CHIKV seropositivity rate was observed in infants (17.6%). Also, most of the dengue positive cases belonged to infants (7.9%). Although the CHIKV seropositivity rate was higher in infants, CHIKV seropositivity was not statistically significant based on the age group (*P* = 0.3). Besides, the observed difference between anti-dengue and age in this study was not significant (*P* = 0.09).

In total, 109/701 (15.54%) in the male group and 101/605 (16.69%) in the female group were positive for anti-IgM CHIKV antibodies and 33/701 (4.07%) in the male group and 49/605 (8.09%) in the female group were positive for anti-IgM DENV antibodies.

The highest dengue seropositivity rate was seen in the female group in youths (logistic regression, OR 1.77, 95% CI 1.11–2.83; *P* = 0.015).

In terms of season, there was a statistically significant difference in the anti-DENV and anti-CHIKV IgM antibody seropositivity rate between the patients grouped based on the season (*χ*^2^ test, *P* < 0.01), and the infection rate in summer was higher than in other seasons (logistic regression, *P* < 0.01).

## Discussion

The seropositive cases in our study reveal possible CHIKV and DENV infection in Iran.

As mentioned before, among the studied provinces, Kerman and Fars had a significant increase in the CHIKV seropositivity rate in comparison with other provinces. Kerman and Fars are located in tropical regions, so these differences can be explained in part by the increasing transmission risk at high temperatures [25]. Another possible explanation for this is that Kerman and Fars provinces are located in southern Iran. Kerman is neighbouring Sistan and Baluchestan, where the presence of *Ae.. Albopictus* and cases of dengue fever and chikungunya fever have been reported. So, it could be suggested that CHIKV has possibly been imported through travellers to this neighbouring endemic country.

The majority of reported dengue cases in previous studies in Iran were from patients who had travelled to endemic countries especially in southeastern Asia. Among them, only one case, unlike previous ones, had been bitten by an infected vector inside Iran [26].

However, some patients had no travel history to dengue-endemic countries [19, 20].Besides, revelations from the only current study done in the Sistan and Baluchistan Province since three decades ago (since 1971), indicated that all the CHIKV^+^ RNA patients had lately traveled to Pakistan according to their records and five of the specimens had analogy with the newly identified cases of the 2016-2017 Pakistani epidemic native to the satellite ECSA genotype of the Indian Ocean descent [21]. Unfortunately, in our study, the travel history of patients is not available.

As a result, the virus may have been spread by patients who have travelled to areas where one or two major vectors, *Ae. aegypti* and *Ae. albopictus* have established. Moreover, an autochthonous silent or paucisymptomatic circulation cannot be excluded and may have found its origin in border countries where vectors are established and autochthonous transmission have been described.

Since 2005, *Ae. aegypti* and/or *Ae. albopictus* have been found in Afghanistan, Algeria, Lebanon, Oman, Palestine, Syria, Turkey and Iran [27]. Although outbreaks of suspected dengue occurred in Pakistan, Yemen, Saudi Arabia, Sudan and Madagascar in 2005–2006, there is no report of autochthonous transmission of DENV from these countries so far [27]. Despite the fact that *Ae. aegypti* is regarded as the primary vector traditionally, some recent epidemics were caused by *Ae. albopictus*, which suggests that *Ae. aegypti* is becoming superseded by *Ae. albopictus* due to virus mutation [28]. *Ae. albopictus* has been distributed widely in Madagascar, Southern Europe and North and South America, Africa, the Indian Ocean and South East Asia via increased international travel and vehicle component such as water-containing tyres imported from elsewhere [28]. For example, vehicles travelling from Turkey, Bulgaria and Syria may introduce *Ae. albopictus* in Iran [15]. A number of factors may have promoted the distribution of Aedes-borne viruses such as urbanisation [29], heavy rainfall [30], war and economic disturbance in Iraq, Syria and Yemen [13], migration [13] and heavy trade in the Red Sea region [30], that may pose risk for spread of DENV from Pakistan to Iran or Afghanistan [19]. With this regard, a significant difference in the high CHIKV and dengue seropositivity rate in summer was observed (*P* < 0.01) in our study. On the other hand, vector mosquitoes are mainly active in the summer season [25]. Based on this finding, we could hypothesise that the vector active season has played an important role in facilitating the autochthonous transmission of imported viruses in Iran. Considering the fact that the presence of mosquito vectors, as well as the infectivity of these vectors, have not yet been studied in Iran since 2014, it is possible that these regions could be the foci of these vectors if the climate conditions were suitable.

Although our study shows no significant difference in the different age groups, however, the prevalence suggests that dengue fever often occurs in children, despite the fact that the older age group has been witnessing an increase in dengue fever infection in recent years [20].

Even though in our study, the presence of the CHIKV IgM antibody is not associated with gender, on the other hand, the anti-dengue IgM antibody is higher in females than in males, which it is not in line with previous reports that exhibited an excess of male patients in Iran [20], Malaysia [31] and Singapore [32]. In line with our study, other researchers have reported a greater proportion of female dengue cases or a slightly differing result of equal proportions of male and female patients in South America [33–36]. The higher dengue fever in males has been attributed to displacements resulting from more business travels executed by males than females in endemic areas [20], while an explanation for the greater frequency of female dengue cases stems from the indoor habits of mosquitoes. Considering the fact that women spend more time at home [37], they are more susceptible to arboviruses.

Given that chikungunya and dengue fever epidemic is engendered by either *Ae. aegypti* and/or *Ae. albopictus,* in a population with low herd or community immunity [28], it is not surprising that Iran may be at high risk for the occurrence of chikungunya and dengue fever epidemic. At this point, it is worth mentioning that the present study is not without its limitations. Firstly, the cross-reactivity between these arboviral infections particularly that of *Flaviviruses,* with other viral infections such as West Nile Virus, which had been previously reported in Iran was not taken into consideration [38]. Another limitation can be explained by the fact that polyclonal stimulation of the immune system or antibody persistence may have had an impact on the specificity of the positive results of the ELISA test. Hence, diagnostic reports should be accompanied by neutralisation assays including the detection or isolation of the virus from patients in order to confirm the presence of these viruses in Iran. The main strength of this study is the large sample size.

## Conclusion

The results from this study suggest that there has been an exposure to DENGV and CHIKV by a local population in Iran. This is an important issue for future research studies and further population-based work is required in order to investigate and assess the degree of exposure of the population to the presence of dengue and CHIKV in Iran.
